# Blue Rubber Bleb Nevus Syndrome with a Novel PDGFRA Variant and Comorbid Autism Spectrum Disorder: A Case Report

**DOI:** 10.3390/children13081009

**Published:** 2026-07-29

**Authors:** Tae Hyeong Kim, Jae Myung Cha, Sung-Hoon Chung

**Affiliations:** 1 Department of Pediatrics, Kyung Hee University College of Medicine, Kyung Hee University Hospital at Gangdong, Seoul 05278, Republic of Korea; pgnkim@khu.ac.kr; 2 Department of Internal Medicine, Kyung Hee University College of Medicine, Kyung Hee University Hospital at Gangdong, Seoul 05278, Republic of Korea; clicknox@khnmc.or.kr

**Keywords:** blue rubber bleb nevus syndrome, *PDGFRA*, variant of uncertain significance, autism spectrum disorder, iron deficiency anemia, venous malformation, genetic testing

## Abstract

**Highlights:**

**What are the main findings?**

- A pediatric patient with BRBNS and severe gastrointestinal bleeding was found to carry a heterozygous PDGFRA variant (c.2075G>T, p.Ser692Ile) of uncertain significance, with no pathogenic variant detected in TEK, PIK3CA, or GNAQ.
- This patient also had autism spectrum disorder (ASD); whether this co-occurrence reflects a biological connection or is coincidental could not be determined from a single case.

**What are the implications of the main findings?**

- A single variant of uncertain significance (VUS), identified from non-lesional tissue in one patient, raises but does not confirm the possibility of genetic contributors to BRBNS beyond TEK.
- These findings are hypothesis-generating; lesional-tissue sequencing, segregation analysis, and functional studies in additional patients are needed before conclusions about genetic heterogeneity or shared mechanisms with ASD can be drawn.

**Abstract:**

**Background:** Blue rubber bleb nevus syndrome (BRBNS) is a rare vascular disorder that primarily affects the skin and gastrointestinal tract, driven predominantly by somatic mosaic mutations, most commonly in *TEK*. Here, we report a case of a child with BRBNS and autism spectrum disorder (ASD). **Case presentation:** A 13-year-old boy diagnosed with ASD at 3 years presented with recurrent abdominal pain, blood in the stool, and severe anemia persisting for 6 months. At admission, his hemoglobin level was 5.6 g/dL. He had received a transfusion at age 6 for unexplained anemia. On examination, he appeared pale but stable, with bluish, compressible nodules on the right index finger and great toe, typical of BRBNS. Laboratory findings were consistent with chronic bleeding-induced iron deficiency. Endoscopic findings revealed multiple vascular lesions in the stomach, duodenum, ileum, and colon. Several colonic lesions were removed and pathologically confirmed as cavernous hemangiomas. Magnetic resonance enterography revealed additional small intestinal lesions. Whole-exome sequencing performed on buccal swab-derived DNA identified a heterozygous *PDGFRA* variant (c.2075G>T, p.Ser692Ile) classified as a variant of uncertain significance; no variants were identified in *TEK, PIK3CA*, or *GNAQ*. The patient underwent endoscopic resection of the larger lesions and received oral iron and a proton pump inhibitor. Hemoglobin stabilized at 11–12 g/dL, and no further transfusions were required. **Conclusions:** This case raises, but does not confirm, the possibility that genes other than *TEK* may contribute to BRBNS. The coexistence of ASD may be coincidental; a mechanistic link remains unproven. Careful endoscopic therapy and medical management controlled bleeding and anemia in this child.

## 1. Introduction

Blue rubber bleb nevus syndrome (BRBNS) is a rare vascular disorder that typically presents during childhood and most commonly affects the skin and gastrointestinal (GI) tract [1]. BRBNS arises predominantly from somatic mosaic mutations in endothelial signaling genes rather than a single germline cause [2]. Cutaneous lesions typically present as soft, compressible blue nodules, whereas GI involvement can range from asymptomatic to recurrent bleeding [3,4]. In most patients, diagnosis of BRBNS is based primarily on characteristic clinical findings. Endoscopic evaluation is often required, sometimes repeatedly, to detect intestinal lesions [5]. In a landmark study, Soblet et al. reported somatic mutations in *TEK*, which encodes the endothelial receptor *TIE2*, in 15 of 17 patients with BRBNS (approximately 88%), confirming the important role of these factors in abnormal angiogenesis [2]. In addition, activating *PIK3CA* mutations have been found in more than half of patients with sporadic venous malformations, further supporting the idea of genetic heterogeneity in vascular anomalies [6]. However, not all patients carry *TEK* mutations, suggesting the involvement of additional genetic mechanisms [7]. Platelet-derived growth factor receptor alpha (*PDGFRA*) encodes a receptor tyrosine kinase involved in angiogenesis and regulation of cell growth [8]. Although *PDGFRA* mutations are well documented in gastrointestinal stromal tumors (GISTs) and inflammatory fibroid polyps, their role in vascular anomalies such as BRBNS remains poorly understood [9]. Recent studies have observed increased *PDGFRA* expression in the cerebellum of individuals with autism spectrum disorder (ASD), suggesting broader developmental implications [10,11]. Here, we describe a pediatric patient with BRBNS, a heterozygous *PDGFRA* variant of uncertain significance, and comorbid ASD, raising the possibility of genetic heterogeneity in BRBNS and a potential overlap between angiogenic and neurodevelopmental pathways, although the variant’s uncertain significance limits any firm conclusion.

## 2. Detailed Case Description

**Figure 1:**
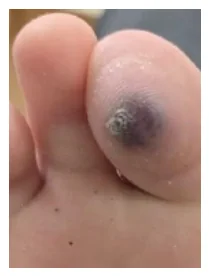
Bluish, compressible vascular nodule on the plantar surface of the right great toe, a pathognomonic cutaneous finding of BRBNS.

**Figure 2:**
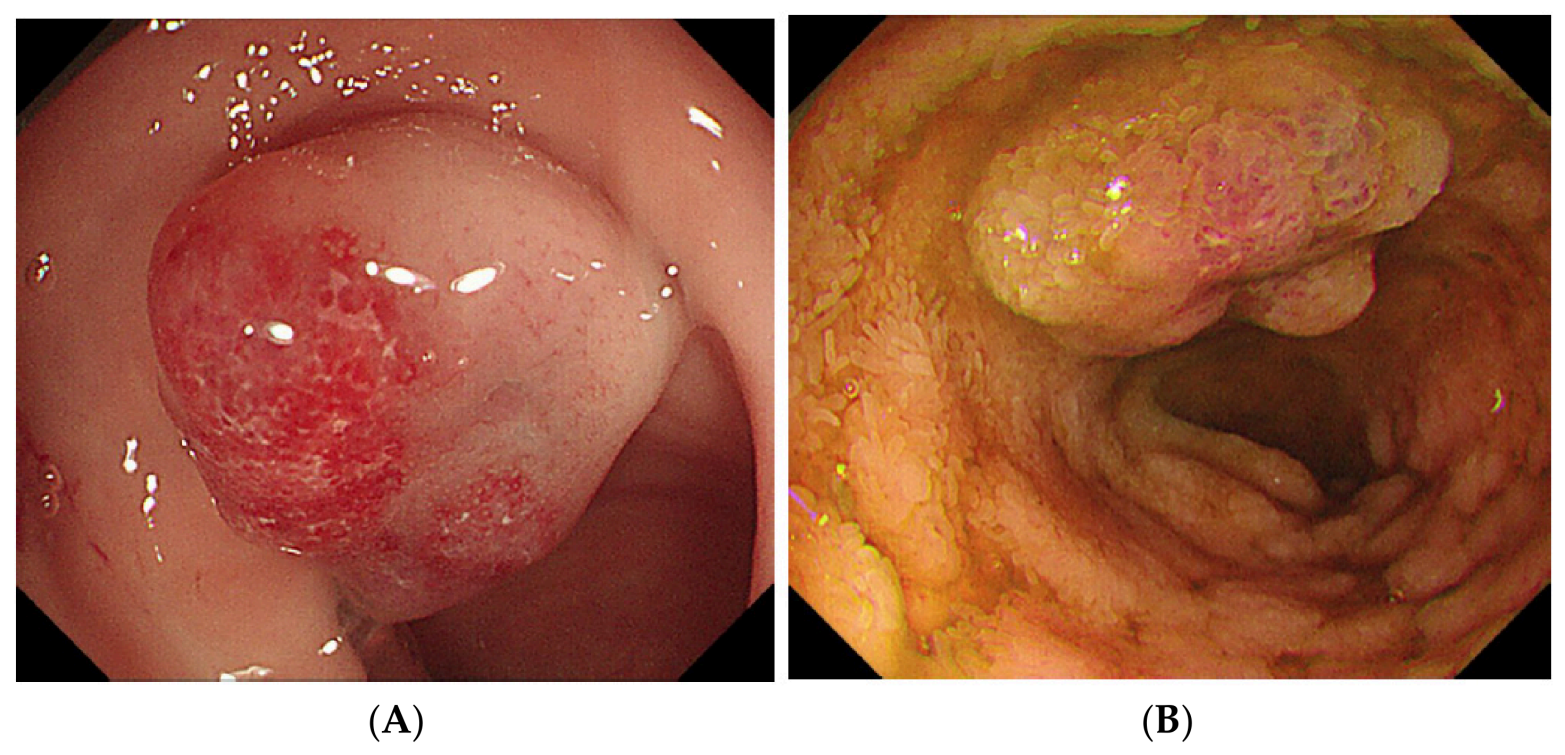
(**A**) Colonoscopic view showing a reddish-blue, compressible vascular lesion in the colon. (**B**) Colonoscopic view showing a similar lesion in the terminal ileum.

**Figure 3:**
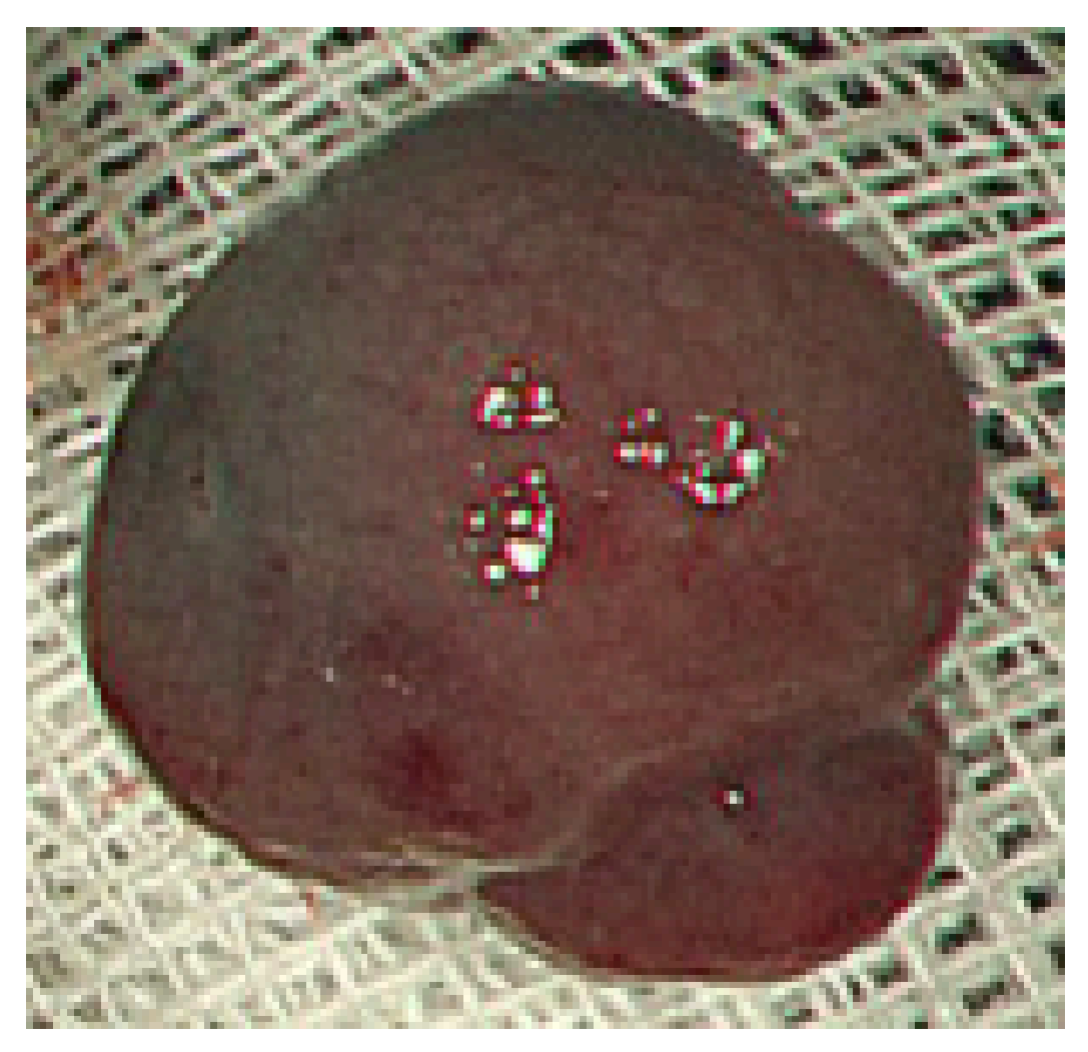
Gross specimen following endoscopic mucosal resection of a vascular lesion from the rectum.

**Figure 4:**
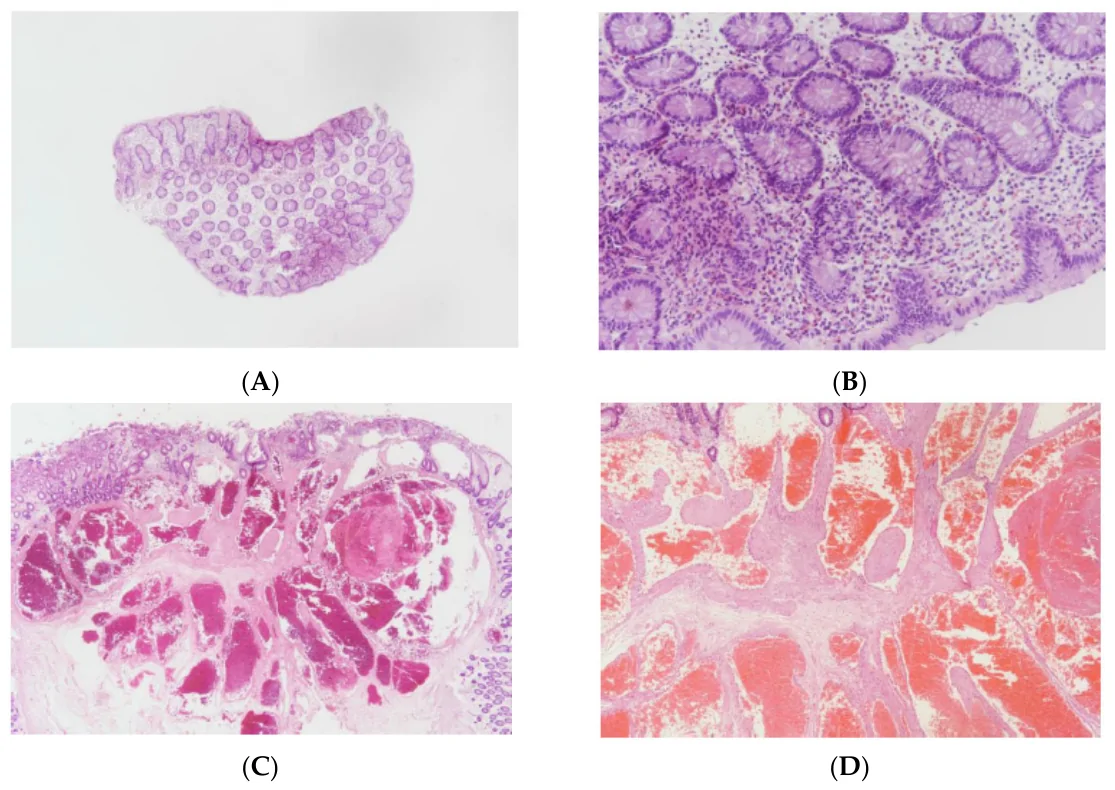
Histopathology of gastrointestinal vascular lesions. (**A**) Cecal lesion (H&E, ×40) and (**B**) cecal lesion (H&E, ×200), showing dilated, thin-walled vascular channels consistent with cavernous hemangioma. (**C**) Rectal lesion (H&E, ×20) and (**D**) rectal lesion (H&E, ×40), showing similar dilated, blood-filled vascular spaces.

**Figure 5:**
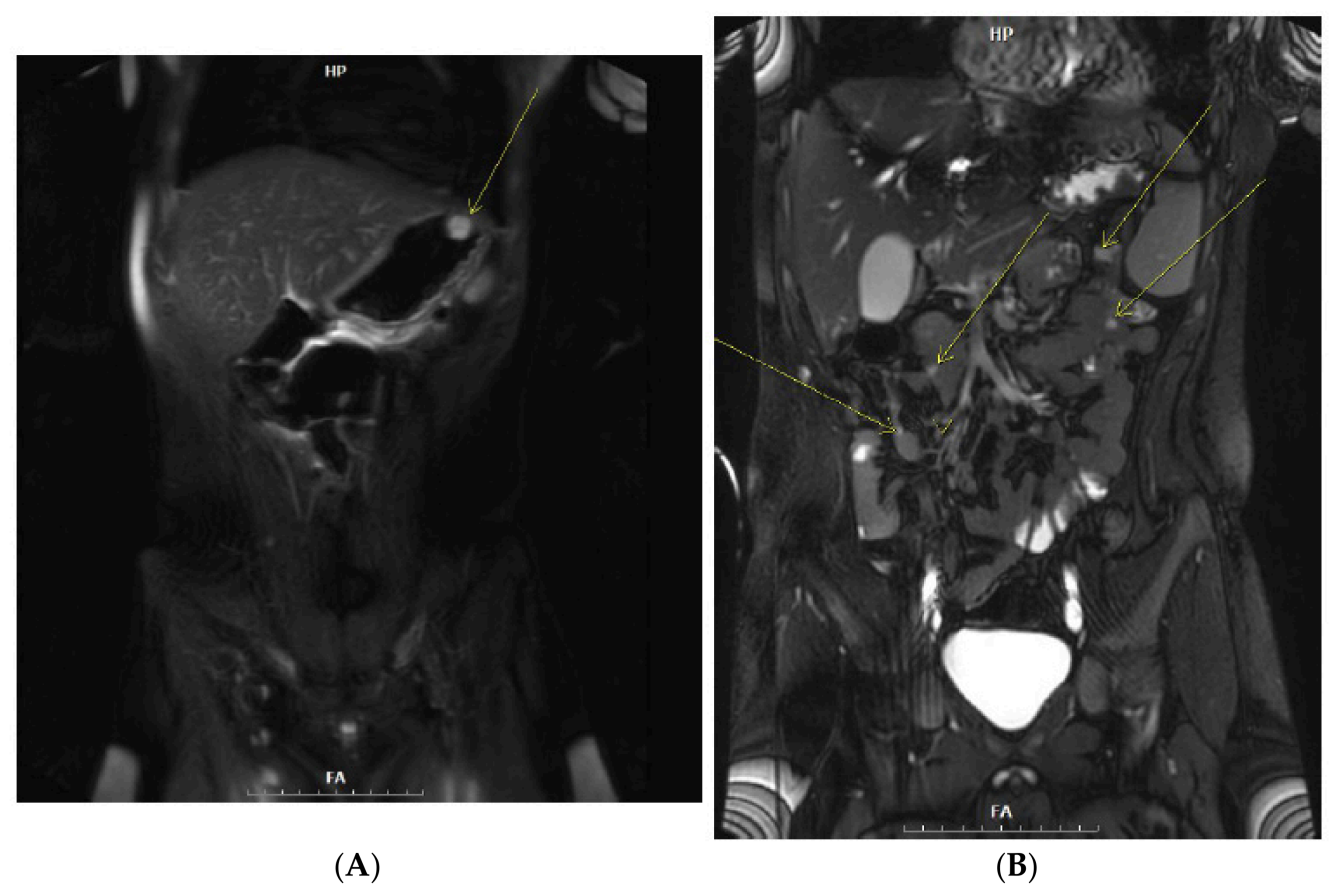
Coronal T2-weighted MR enterography. (**A**) A small hyperintense polypoid lesion in the gastric fundus (arrow), consistent with a venous malformation. (**B**) Additional hyperintense small bowel lesions of varying size (arrows), indicating involvement not fully captured by endoscopy.

A 13-year-old boy presented to our pediatric clinic with a 6-month history of recurrent abdominal pain and intermittent hematochezia. He had been diagnosed with ASD at 3 years of age based on Diagnostic and Statistical Manual of Mental Disorders (DSM-5) criteria and had received special education and behavioral therapy. At 6 years of age, he received a red blood cell transfusion for severe anemia of unknown etiology. There was no family history of vascular malformations or bleeding disorders. On physical examination, the patient appeared pale but was hemodynamically stable. Dermatological assessment revealed pathognomonic lesions of BRBNS, including a 12-mm soft, bluish, compressible nodule on the right index finger and a similar 15-mm lesion on the plantar surface of the right great toe (Figure 1). Laboratory evaluation revealed severe microcytic anemia (hemoglobin, 5.6 g/dL) and a very low ferritin level (2.5 ng/mL), suggestive of chronic blood loss. Mild leukopenia was present, with a white blood cell count of 3.8 × 10^3^/μL, which was not further evaluated as it was not considered clinically significant, whereas the platelet count was within the normal range. C-reactive protein and fecal calprotectin levels were unremarkable, and stool occult blood testing yielded intermittently positive results, consistent with the patient’s known GI lesions. Endoscopy revealed multiple compressible, reddish-blue vascular lesions scattered throughout the stomach, duodenum, terminal ileum, and colon (ranging from 15 mm to 25 mm in diameter; Figure 2). Several accessible colonic lesions were removed by endoscopic mucosal resection (Figure 3). Histopathological examination of the resected specimens confirmed the presence of cavernous hemangiomas composed of dilated, thin-walled vascular channels lined with flattened endothelial cells without atypia (Figure 4). Coronal T2-weighted MR enterography demonstrated a small hyperintense polypoid lesion in the gastric fundus (Figure 5A, arrow) and several additional hyperintense small bowel lesions of varying size (Figure 5B, arrows), indicating more widespread GI involvement than was accessible by endoscopy alone. Whole-exome sequencing (NovaSeq X, Illumina; IDT xGen exome panel, Integrated DNA Technologies), performed on DNA extracted from a buccal swab, achieved a mean coverage of 111× (≥20× in 99.6% of target bases). Variant calling and classification were performed via the EVIDENCE v4.3 platform [12] (GATK v4.4.0, Manta v1.6.0, VEP v104.2) per ACMG/AMP guidelines [13], revealing a heterozygous missense variant in *PDGFRA* (c.2075G>T, p.Ser692Ile) in exon 15, present at a variant allele fraction of 54% (91 of 167 reads at this locus). This substitution affects a serine residue within the tyrosine kinase domain. In ClinVar (VCV001408376), the variant was listed as having uncertain significance, catalogued in association with GIST/GIST-plus syndrome (OMIM: 175510) rather than BRBNS specifically, and was not observed in the gnomAD v4.1.0 population. In silico predictions were inconclusive (REVEL 0.378; 3Cnet 0.02, a low score not supportive of pathogenicity). No pathogenic variants were detected in the *TEK* gene, *PIK3CA*, or *GNAQ*. As testing was proband-only, without segregation analysis or Sanger validation, whether this variant is somatic or germline could not be determined. The patient recovered without complications. The patient was started on oral iron supplementation and a proton pump inhibitor. During the 6-month follow-up period, hemoglobin levels gradually improved and stabilized between 11.0 and 12.0 g/dL, with no further transfusions required. He experienced occasional mild abdominal discomfort but no recurrent episodes of significant bleeding. Regular endoscopic surveillance was performed. 

## 3. Discussion

To the best of our knowledge, this is among the first pediatric cases of BRBNS associated with a *PDGFRA* variant in the absence of a *TEK* mutation. This finding is consistent with the genetic heterogeneity of BRBNS beyond the *TEK* pathway, although this observation is based on a single variant of uncertain significance and should not be considered confirmatory [2,14]. *PDGFRA* encodes a receptor tyrosine kinase involved in the regulation of angiogenesis, cellular proliferation, and tissue growth [15]. Our patient harbored a p.Ser692Ile substitution in the tyrosine kinase domain, specifically within the catalytically active region [16]. In silico predictions for this variant were inconclusive: the REVEL score (0.378) fell in an intermediate range, while the 3Cnet score (0.02) did not support pathogenicity, consistent with its classification as a VUS rather than a likely pathogenic variant. In other clinical contexts, *PDGFRA* mutations are best known in GISTs and occur in approximately 5%–10% of cases [17]. Because DNA was obtained from a buccal swab rather than affected lesional tissue, we cannot definitively determine whether this variant is causally related to this patient’s vascular phenotype. The variant was present at a variant allele fraction of 54% in non-lesional tissue, a proportion consistent with a constitutional heterozygous variant present throughout the body rather than with low-level somatic mosaicism confined to a subset of tissues. BRBNS-associated pathogenic variants are typically somatic and lesion-restricted, arising from postzygotic mutation in endothelial precursor cells [2]; the constitutional-range allele fraction observed here is also compatible with a coincidental finding, and confirmatory sequencing of affected lesional tissue was not performed to establish a tissue-specific origin. No segregation analysis or parental testing was performed. Given these substantial uncertainties, we do not consider genetic testing of first-degree relatives to be indicated on the basis of this single proband-only VUS.

The co-occurrence of BRBNS and ASD in this patient may point to shared molecular pathways underlying these two conditions. Recent transcriptomic data have shown increased *PDGFRA* expression in the cerebellum of individuals with ASD [10]. Additionally, whole-genome transmission disequilibrium testing has revealed associations between *PDGFRA* and sensory-related behavioral phenotypes in ASD [11]. Furthermore, mouse models of maternal immune activation have demonstrated altered *PDGFRA* expression associated with ASD-like behaviors [18]. Although the current case does not establish a causal relationship, it suggests *PDGFRA* signaling could contribute to both vascular and neurodevelopmental phenotypes. However, a causal relationship in humans remains unproven, and this co-occurrence may be coincidental.

In children with BRBNS, treatment should aim to control recurrent GI bleeding while minimizing procedure-related risks. In our patient, endoscopic mucosal resection effectively controlled bleeding from accessible colonic lesions, consistent with recommendations favoring endoscopic intervention when feasible [19,20]. Given the patient’s clinical stability following endoscopic resection and iron supplementation, conservative management was chosen over systemic therapy, such as sirolimus, which is used for more complicated vascular anomalies [21]; the patient did not receive sirolimus or any other systemic agent, and this option will be reconsidered if new lesions or recurrent bleeding occur. Iron-deficiency anemia is one of the most clinically significant complications of pediatric BRBNS and requires timely and aggressive management to prevent adverse neurocognitive outcomes [22]. Our patient’s good outcome with oral iron administration and endoscopic intervention demonstrates that a staged, multimodal strategy can be effective for managing anemia in pediatric patients with BRBNS. 

Our study has certain limitations. The PDGFRA variant is a VUS identified from non-lesional (buccal swab) tissue only, without segregation or orthogonal validation data. The in silico predictions and allele fraction are informative but do not establish pathogenicity or a tissue-specific somatic origin. This variant has not been functionally studied; therefore, its pathogenic role remains unknown, and any connection with ASD cannot be proven in a single case. Further patient and laboratory studies are needed to determine whether *PDGFRA* is involved in BRBNS and whether it is related to neurodevelopment.

## 4. Conclusions

In conclusion, we report a pediatric case of BRBNS associated with a *PDGFRA* variant and comorbid ASD. This case is consistent with genetic heterogeneity in BRBNS; however, this observation from a single VUS is hypothesis-generating and requires confirmation in additional cases. The coexistence of BRBNS and ASD may suggest shared biological pathways; however, further investigation is needed to clarify this potential association. Our patient responded favorably to endoscopic and conservative therapies, supporting a practical approach that combines early detection and multidisciplinary care.

## Data Availability

The datasets used and analyzed in this study are available from the corresponding author upon reasonable request, subject to patient privacy and confidentiality requirements.

## Abbreviations

ASD	autism spectrum disorder
BRBNS	blue rubber bleb nevus syndrome
GIST	gastrointestinal stromal tumor
PDGFRA	platelet-derived growth factor receptor alpha
TEK	tyrosine kinase receptor (TIE2)
VUS	variant of uncertain significance
